# Mercury, Blue-Pill, & Calomel—Their Use and Abuse

**Published:** 1840-04-01

**Authors:** 


Mercury, Blue-pill, and Calomel: their Use and Abuse. By *G. G. Sigmond*, M.D. &c. &c. Lecturer on the Practice of Physic at Sydenham College, &c. &c. Duodecimo, pp. 129, Renshaw, 1840.

The Author of this little volume is one of the now *comparatively* few physicians who combine not only classical literature, but the literature of their profession, with the science and practice of it. Dr. Sigmond has paid great attention to certain branches of his profession which used to be considered auxiliary, but are now wisely deemed integral portions of medicine—namely, botany and chemistry. Pharmacology, too, which is still more essential than the others, has been carefully cultivated by our author. In the present volume he has selected one of the most important—if not *the* most important, articles of the materia medica, for dissertation, and has handled it with great ability. He modestly claims the merit of compilation, rather than of composition; but it is very evident that there is much of the latter in this volume. And were it otherwise, the judgment necessary for careful selection, and the graces of style calculated to render science attractive, are no trifling merits. Dr. S. justly observes that, his " difficulty has been to condense the vast mass of materials which the judgment, the profound knowledge, and the labour of a great number of writers in different languages, have amassed, and to place it in an attractive form before the young and inexperienced."

The history of mercury, from the times of alchymy, down to our own days, is traced with great labour and research—then the particular preparations of this metal are noticed, and the opinions of eminent practitioners adduced for and against them. The following quotation shews the practical turn of our author's observations.

" That which is commonly considered a most innocent medicine may be the source of the utmost harm, if it be taken at an improper moment, or under unfavourable circumstances. Medicines generally and apparently unhesitatingly prescribed, are given with safety by those only who are intimately acquainted with their modus operandi. Thus magnesia, than which nothing can be more useful under proper regulations, and nothing is considered more simple, has been productive of fatal consequences, from the ignorance with which it has been administered, or the perseverance in taking it when it has failed in its expected influence. Masses unchanged have been found after death closely collected together, or patches of the powder adhering with the utmost pertinacity to the intestines, because there had been none of the acid with which it should combine to be properly efficacious. Some very curious instances of this kind are upon record, and some of the cases have been, from the apparently suspicious circumstances, made subjects of legal investigation; for even death from arsenic has been supposed to have taken place, when examination has shown that magnesia has been its cause. Manna, so useful a laxative to children, is not to be used incautiously, notwithstanding its usual harmlessness. When much vegetable food has been taken, more especially in young children, if this remedy be given, dyspepsia of a most aggravated character occurs; the quantity of flatulence produced has been a fearful evil, and the consequences have been alarming. Castor oil, one of the favourite popular remedies, if given under improper circumstances, will not only occasion excruciating tormina, but will be the cause of the expulsion of the mucus which lubricates and defends the passages from injury, and what have been supposed to be exfoliations have taken place, leaving behind a surface so irritable, that months have elapsed before a normal state has prevailed. The neutral salts, those of Epsom, of Cheltenham, or of Harrowgate, are not to be trifled with; and many individuals who have recourse to them without proper advice, have to repent that folly; diarrhœa, dysentery, and sometimes dropsy, supervene upon their injudicious use. Gamboge, which has lately crept into fashion as a purgative, is of all others the most uncertain, and oftentimes the most pernicious; its influence is principally exerted upon the muscular fibre, and hence peristaltic action is increased; and as there are many who, from want of proper attention, have costiveness dependent upon a sluggishness of action, they find benefit from pills in which this forms a principal ingredient. Its power, when it is properly exerted, is very striking, and it becomes, in the hands of the well-informed man, a very valuable adjunct, but it is a most energetic engine of mischief; it has been known to produce introsusceptio, having, from the vigour of its action, caused an inverted motion, such are its stimulating power upon the muscles; and in some of those instances which have come before the public, of death produced by violent action of pills upon the intestinal canals, this drug has decidedly been the means employed. The good sense of the public has taught it to give up the constant employment of aloes, once the basis of every pill that was to act upon the bowels, and gamboge, which is infinitely more mischievous, has unfortunately been substituted; but, of the two evils, the hæmorrhoids produced by aloes are infinitely preferable to the diseases and to the results consequent upon the other purgative. Even senna, the valuable ingredient of the black draught, and which certainly comes nearer to a harmless domestic remedy than any other, is not so alone; it will disorder the smaller intestines for a great length of time; it is not only a momentary cause of griping and of inconvenience, but it leaves behind it a very great tendency to those uncomfortable sensations, and more particularly if the liver have not been previously called into some slightly increased action, by which the bile is poured forth, and thus the general action of the intestinal canal be duly and properly augmented; these circumstances demand the very greatest caution and attention. Indeed, a catalogue of sorrows occasioned by the indiscriminate and foolish use of purgatives might be drawn up; but such is the headstrong tendency some have to doctor themselves, that it would be rather a curious than a useful task to undertake it." 60.

Dr. S. after noticing the revolution effected in the treatment of fever by calomel purgatives in the hands of Dr. Hamilton,—as also in chlorosis, hysteria, chorea, &c. observes:—

"It has been stated upon good authority, that the putrefying mass, emitting a faint, nauseous, and exceedingly pungent smell, which has been first expelled, after medical assistance, from constipated habits after febrile disease, is capable of transmitting infection to a very considerable distance, and constitutes a miasm of the most fearful character, which speedily depresses the nervous energy of those who may be thrown within the reach of its influence; and hence spring up the host of fevers, which are familiar to some countries, spreading with the most fearful rapidity, and attacking most especially those who are by want of care predisposed to every exciting cause of disease. Few depressing agents are more instantaneous or more certain than the effluvia issuing from fæcal matter, and sometimes in hospitals it has been found that ulcers put on their most unfavourable aspect, if the suffering patient be in the neighbourhood of the temple of Cloacina." 65.

He properly remarks that the system of purgation has been carried too far by some practitioners, notwithstanding the checks sustained by the unreasonable dread of aperients entertained by the disciples of Broussais, and by the Continentals generally.

Dr. Sigmond's description of the catharto-mania is very humourous—perhaps a little too highly-coloured—but still within the boundaries of verisimilitude.

"It has been very sagaciously observed, that the public are naturally fond of the doctrine of the humoral pathologists, and that they are most willing to believe, that, by active purging, there is carried off a great deal of poisonous matter which would enter into the system, and gradually overpower it. When a medical man is called in who is an advocate for the purging system, patients generally soon fall into his way of thinking; they have a sort of gratification in discharging a large quantity of nasty-looking, greenish or black stuff; and if the excretion be attended with any marked fetid odour, they feel a sort of selfcomplacency; they fancy they have discharged a mass of corruption, and they are very far from being indisposed to the advice of the learned doctor who recommends them another dose of blue-pill and a draught; they say they are quite ready for it; and are quite pleased at the idea of another disgorgement, and cannot resist the gratification of making another attack upon the enemy, who, they fancy, must very speedily collect his forces. They voraciously swallow the doctor's stuff, and, as they say, they find themselves lighter, and their appetites improve. They are very much pleased with what has been done for them; and, indeed, were they then to rest contented, all would be well; but they fancy they have discovered the means of attaining lasting health, they therefore give way to the pleasures of the table, quite satisfied with the idea that they know how to get rid of any accumulation that may occur, and think that they are perfectly capable of taking care of themselves; then probably once or twice in the course of every week they swallow some drastic purgative, either quite ignorant of what they are employing, or, from having found, under the hands of the doctor, such relief from calomel or blue-pill, they take a fixed dose of one or other of these. Sometimes, after dinner, a kind friend recommends his own pill from a prescription he had from Abernethy or Baillie, or the fashionable dyspeptic doctor of the day, which he declares he has used twice a week for the last five years, and never known an ache or pain since; the greedy auditor swallows the tale, and afterwards the physic, whose composition may be completely opposed to his state; he goes on with it, week after week, disordering his bowels, expelling the natural mucus, urging on the canal to increased action, till at last his stomach and the whole digestive apparatus become really impaired. He then goes from physician to physician, all over London: each gives him a prescription for a tonic, a stimulant, or a bitter; at length a permanent irritation is kept up, the whole system sympathises with the state of the alimentary canal, the mind is feverish and irritable, the viscera become deranged, and at last the poor sufferer falls a victim to the early benefit which he had received from the proper administration of medicine, and then from its abuse." 67.

"The unnatural colour and the fetid odour of the stools, are quite as often produced by the medicine that has been exhibited, as by any other cause; for often does calomel give rise to the very odour which some persons think is a proof that medicine is to be continued; the slimy stools of children, which are often the cause of purging being carried on to a very great extent, are, in many instances, the consequence of what has been given. As long as the remedial agent is taken, the fæcal residue does not acquire its wonted solid consistence, nor give forth the usual smell, which is acquired from its remaining in a certain portion of the large intestines. It is but, comparatively speaking, from a limited surface that the mixed odour is acquired, and there is a very great difference between that which is caused by the secretion from the liver, and that by the lower portion of the intestinal canal. The offensiveness of the fæcal evacuation, though it may guide the practitioner as to the particular viscus which is disordered, cannot serve as any assistant by which he may ascertain whether more action is required." 68.

In weakly children, the system of purgation is often injurious, by carrying off nutriment before the chyle is extracted from it. It is not, as Dr. Sigmond has judiciously remarked, on mere purgation that the utility of purgative medicine depends; but upon their exciting the salivary, gastric, biliary, pancreatic, and other secretions, and thus aiding the functions of digestion and assimilation. We may observe, from very long experience, that, while the motions are dark and fætid, *without mucus*, there is little danger from purgation, where other symptoms appear to require that measure; but whenever the natural mucus of the bowels begins to present itself with the motions, we ought to slacken our pace, or much mischief will be done. It is curious, however, that patients, in general, think themselves very lucky in getting rid of mucus, or "slime," as they suppose it acts injuriously in the bowels, whereas it is the removal by medicine of that which ought to remain.

Dr. Sigmond glances lightly but historically, at the various writers who have directed the use of mercury to the cure of diseases, and presents us with a curious coup d'œil of the clashing opinions and testimonies of different authors, respecting this celebrated mineral. We quote the following passage as deserving attention.

"Minute doses of mercury frequently repeated have met with their warm advocates both in chronic and acute diseases; and when Abernethy's mode of administering blue pill has been unsuccessful, the plan of diminishing the dose, and increasing the frequency, has been inculcated. From half a grain to the eighth of a grain has been proposed, and has been considered beneficial, given at intervals of from six to eight hours. I have seen great irritation of the system kept up by this treatment in many cases in which this plan has been followed. In affections in which a vitiated state of the bile was predominant, a degree of nervous excitement has been often produced upon the third or fourth day, which has called for a discontinuance of the remedy. Notwithstanding a rigid attention to the rules which have been laid down in the management of small doses, I have had occasion to observe failures where benefit was expected to be derived. Nor have I found any cases in which Abernethy's system failed from any of the causes which usually operate, in which blue pill could be borne in any dose. I have uniformly found, where blue pill was distressing in one dose, that it was equally so in another, and I have rarely found the minute quantity better borne than the larger, if repeated. An active dose of medicine has generally been absolutely necessary to carry off the source of irritation, and sometimes nervous depression. A great tendency to action, on the mucous membrane especially, marked by sore-throat, has speedily followed a perseverance in this system. To give tone and strength to the system during mercurial medicines generally requires great knowledge of the patient's constitution; for irritation and excitement are as often the result as the depression and debility, which indicate the necessity for the employment of these powerful agents." 89.

The author makes many sensible and practical remarks on sarsaparilla, which we recommend to the young practitioner.

The following passage is deserving of quotation.

"A distinction is to be recognised between laxative and purgative medicines: the first merely emptying the bowels of such fæcal contents as may already be lodged there, whilst the second class have the power of inducing a still further increase of the quantity, and of stimulating the vessels to pour forth more excrementitious matter. The chloride of mercury will, according to the dose in which it may be given, produce either of these effects; but it is the biliary secretion that is most augmented, and this is particularly marked by an alteration in the colour and the odour of the fæces. All the preparations of mercury, more or less, have this influence; and patients who have, for a length of time, excreted only blackish and unhealthy looking dejections, discharge them of a yellow hue, and of a different odour, after a few grains of calomel or of blue pill. The odour is oftentimes rendered more disagreeable, and a change takes place in the gases that are developed. The colour depends upon the kind and quantity of the bile secreted by the liver; and where any obstruction takes place, the stools are pale or whitish. The bile itself is of a deep yellow brown colour, and, as Abernethy has observed, it is like wetted rhubarb; if either of these substances be put into a large quantity of water, they will dye it of a bright yellow colour, which is actually the colour of these substances, yet it is so concentrated in the mass as to appear of a deep brown. Green bile is ordinarily the result of disordered function, although it has been occasionally found in the gall-bladder, where the liver is in a perfectly healthy state. Vegetables frequently give their colour to the fæces. The peculiar fætor of the residue of the alimentary matter is acquired in the large intestines; and if the small intestines at their termination, and the large intestines at their beginning, be examined, there will be found almost a line of demarcation. To what this is owing we are at a loss to say. It is not alone a chemical decomposition that occurs, but the animal economy imparts a peculiar change. The examination of the fæces has been but little prosecuted: although a subject of some interest, it is one that requires great enthusiasm in the cause of science, and very little delicacy in the sense of smell; nay, the very perusal of some authors' inquiries is nauseating.

Lavoisier wrote a memoir on the nature of the aeriform elastic fluids, which are disengaged from certain animal substances during fermentation; his experiments prove that human excrement emits, at the temperature of 60 degrees, a gas, a mixture of eleven parts carbonic acid gas, and one part of an inflammable gas, which burnt with a blue flame, and was, therefore, probably carbonic oxide gas; the experiments of Thenard and Dupuytren prove, however, that sulphuretted hydrogen is likewise exhaled from human fæces collected in large quantities; the changes that take place out of the body, by the action of the humid air, appear to be very rapid. Fresh fæces do not effervesce with dilute sulphuric acid, but old moist fæces do, emitting the volume of carbonic acid gas which they do at first. The chloride of mercury appears to exercise over the action of the large intestines some power, as the gaseous exhalations materially differ when it has been employed; and this is particularly striking in children, the odour of the dejection being very materially influenced in them, and much more offensive fætor being the result; and this, in general, is a proof of the due action of the medicine, for the system, previously disordered, appears thus to rid itself of a deleterious agent.

Whether the extrication of the different gases be natural to the human body, or whether it be the result of our artificial habits of life, I will not presume to say. It is stated that our living on animal food is the cause of the greater number of the diseases to which man is subject, and that it also imparts to that which is excreted from man the offensive odour: and certainly many animals that are herbivorous are inoffensive on that point, whilst carnivorous animals are the reverse; and some philosophers have entered upon some curious speculations arising out of these circumstances. Rousseau, indeed, draws a conclusion, that man is not calculated for the social state, because his excretions, his effluvia, and his emanations, are destructive to his fellow-creature. Whatever may be the means by which the inflammable air is extricated from the intestines, certain it is that great indications of the nature of the matter taken in by the mouth, and its effects upon the health, may be drawn from a knowledge of the odour evolved, and from correcting it by the administration of medicine. Disease commencing in the rectum and large intestines, may often be recognised by the quantity of flatus evolved, and unnatural diet will keep up an irritation which may prove very injurious." 109.

The work winds up with a dissertation on the injurious effects of mercury imprudently exhibited; with some most judicious rules to be observed during the administration of mercurial preparations. We think we have said enough, and exhibited sufficient specimens of this little volume, to insure it a favourable reception with all those who have not possessed the original lectures. We hope the author will pursue his researches and experiments on the vast catalogue of medicinal agents—a field most extensive, and for the cultivation of which Dr. Sigmond possesses excellent qualifications.

